# Improving Medical Assessment Validity with Generative AI: Lessons from Human–AI Interaction in Bloom’s Taxonomy Classification

**DOI:** 10.1007/s40670-026-02719-2

**Published:** 2026-04-18

**Authors:** Rocicleide de Lima Lopes, Marcos Kubrusly, Juliana Silva Arruda, Arnaldo Aires Peixoto Junior, Marília Marques Guimarães, José Lima de Carvalho Rocha, Hermano Alexandre Lima Rocha

**Affiliations:** 1 Christus University, R. João Adolfo Gurgel, 133 - Cocó, Fortaleza, CE 60190-180 Brazil; 2 Community Health Department, Federal University of Ceará, R. Papi Júnior, 1223 - Rodolfo Teófilo, Fortaleza, CE 60430-235 Brazil

**Keywords:** Bloom's Taxonomy, Generative AI, Medical education, Assessment validity, Human-AI interaction

## Abstract

**Introduction:**

While Bloom's Taxonomy is essential for developing higher-order clinical reasoning, its practical application by faculty remains limited. This perpetuates a reliance on assessments that favor memorization over clinical reasoning. This study aimed to develop and validate a Generative AI tool to assist educators in accurately classifying assessment items, and to evaluate the tool's impact on medical faculty's classification accuracy.

**Methods:**

A two-phase experimental design was used. Phase 1 involved training and validating a ChatGPT-4 model using 200 medical exam items classified by a panel who established a “gold standard”. The model’s performance was then tested on an independent set of 100 items and assessed using overall accuracy, Cohen’s Kappa, and Matthews Correlation Coefficient (MCC). Phase 2 involved experienced medical professors using the validated AI tool.

**Results:**

In Phase 1, the AI model achieved a high overall accuracy of 95.0% (95% CI: 90.0–99.0%) and “very good” inter-rater agreement with the expert standard (K = 0.85). In Phase 2, faculty demonstrated high overall adherence (75.2%) to the AI’s recommendations. In cases of disagreement where faculty chose to override the AI, the faculty’s final decision was accurate only 29.4% of the time, demonstrating human overconfidence.

**Discussion:**

Generative AI, despite a specific and predictable flaw in differentiating “Analyze” from “Understand,” serves as a powerful partner for medical faculty. The tool provides a more reliable classification than un-aided, non-expert human judgment. It can significantly improve assessment validity by supporting faculty, helping to bridge pedagogical training gaps, and promoting the development of assessments that target higher-order cognitive skills essential for medical practice.

## Introduction

The increasing complexity of assessment processes in medical education has revealed a central paradox: although Bloom’s Taxonomy is widely recognized as an essential instrument for promoting cognitively higher-level evaluations, its practical application remains limited by human and structural factors. Originally conceived by Benjamin Bloom [1] and later revised by Anderson and Krathwohl [2] the taxonomy proposes a hierarchy of cognitive skills: remember, understand, apply, analyze, evaluate, and create, which guides the development of educational objectives and the formulation of assessment instruments aligned with progressively higher levels of mental complexity. However, despite its theoretical importance, teachers, even those with extensive clinical experience, often face difficulties in operationalizing this structure into consistent and valid assessments. This methodological gap not only compromises the pedagogical validity of examinations but also perpetuates a teaching culture centered on memorization and information reproduction, to the detriment of clinical reasoning and reflective decision-making. According to Luckesi [3] educational assessment is not merely an instrument for measuring learning outcomes, but a pedagogical act intrinsically linked to decision-making and the promotion of meaningful learning. Rather than serving as a mechanism of control or classification, assessment should function as a continuous and reflective process that guides both teaching and learning. From this perspective, the development of educational objectives and assessment items gains renewed importance, as it determines the coherence between what is taught, how it is taught, and how students’ knowledge is evaluated. Within this framework, Bloom’s Taxonomy offers a structured reference for aligning cognitive complexity with pedagogical goals, ensuring that assessment transcends memorization and stimulates higher-order thinking skills essential to medical education.

The development of items in objective examinations is a central part of the educational process, as it guides pedagogical planning, teaching, and student learning. In this context, Bloom’s Taxonomy remains one of the most widely used references for structuring educational objectives and classifying cognitive skills into progressive levels, ranging from the recall of simple facts to the creation and evaluation of new knowledge [2]. The application of taxonomy ensures that assessments are not restricted to memorization, but favor the development of higher-order skills, such as clinical reasoning, complex case analysis, and critical judgment, which are fundamental aspects of medical training [4, 5] .

Despite its widespread use, teachers in different areas face difficulties in formulating and classifying items according to Bloom’s levels, either due to the lack of structured pedagogical training or the inherent complexity of aligning objectives, content, and assessment strategies [6, 7]. This challenge is even more evident in medical education, where many teachers enter the profession based on their clinical experience, without formal training in the construction of robust and reliable assessment tools, where the physician becomes a teacher by signing a contract with institutions or passing a competitive exam, but not because they have been prepared for this role [8]. As a result, assessments focused on lower cognitive levels prevail, limiting the stimulation of analytical and clinical decision-making skills.

To address these challenges, we must look beyond the mechanics of testing to the theoretical foundations of assessment validity and learner cognition. Contemporary validity frameworks, such as those proposed by Messick and Kane, emphasize that validity is not an inherent property of a test, but an argument supporting the interpretation of scores. In this context, the accurate classification of items according to Bloom’s Taxonomy provides essential content evidence (Messick) and supports the scoring inference (Kane). If an item intended to test ‘Analysis’ actually functions at the ‘Remember’ level, the validity argument collapses; the score no longer represents the student’s ability to reason clinically, but merely their ability to recall facts [9, 10]. 

Furthermore, Cognitive Load Theory (CLT) warns that misaligned assessments impose extraneous cognitive load. When students encounter assessment items that are methodologically flawed or misclassified—appearing complex but requiring simple recall, or vice versa—their working memory is taxed by the need to decipher the assessment logic rather than processing the clinical problem. This ‘bad difficulty’ interferes with the measurement of intrinsic load, which represents the actual complexity of the medical material. By utilizing Generative AI to verify that item complexity matches the intended cognitive level, educators can ensure that the assessment load aligns with the learner’s developmental stage, thereby fostering fairness and pedagogical alignment [11]. 

Artificial Intelligence has emerged as a powerful force for transforming the way we teach and learn [12]. Machine learning models and, more recently, deep learning models have been applied to the classification of learning objectives and assessment test items, achieving promising but still limited performance at more complex cognitive levels, with accuracies often below 90% [13, 14]. The introduction of Generative Language Models (LLMs), such as ChatGPT, represents a substantial advance, given their ability to understand natural language in broad contexts and offer contextualized responses. The processing of this natural language covers various tasks/areas such as “voice-to-text, translation; improvements in education; clean and cheap energy; fraud detection; safer (transportation apps), faster (optimized routes), and cleaner means of transportation“ [15]. There are already experiences of using these models to address Bloom’s taxonomy, but they are disconnected from educational practice and teachers and have unsatisfactory results that “reveal clear inconsistencies in relation to expected human progression” [16] .In this context, the research question is: How can a Generative Language Model [17] assist teachers in accurately classifying assessment items according to Bloom’s Taxonomy?

The use of AI systems has been increasingly sought after in educational processes, but it requires continuous validation of its accuracy, since incorrect recommendations can lead to misinterpretations by human users [18]. Cognitive phenomena such as *anchoring bias* and automation bias can lead teachers and evaluators to uncritically accept the first algorithmic suggestion, even when incorrect, shifting their subsequent judgments, and studies evaluating these aspects for Bloom’s taxonomy with LLM do not yet exist. Thus, in this study, we aim to develop an LLM application to assist teachers in classifying Bloom’s taxonomy and evaluate the results of teachers’ use of this tool.

This research is personally justified by the commitment to improving fairer and more formative assessment practices in medical education, a field in which teaching often lacks structured pedagogical support. Academically, it contributes to advancing knowledge by integrating artificial intelligence and educational theory, promoting methodological innovation. Scientifically, it provides evidence on the validity and applicability of generative models in cognitive classification. Socially, it aims to positively impact the training of more critical, reflective, and well-prepared health professionals, thereby strengthening the quality of education and healthcare delivery.

## Methods

### Study Design

This study used a quantitative and experimental approach to evaluate the performance of a model based on Generative Artificial Intelligence [17] in classifying test items according to the modified Bloom’s Taxonomy. Initially, in phase 1, the model was trained and refined using a dataset previously classified by teachers and a specialist (training set). Subsequently, the performance of the refined model was evaluated on an independent test set containing 100 test items classified by experts (validation set). Finally, in phase 2, a GPT application was generated with the generated model, which was tested by medical school professors with experience in medical education using a different set of items.

**Fig. 1:**
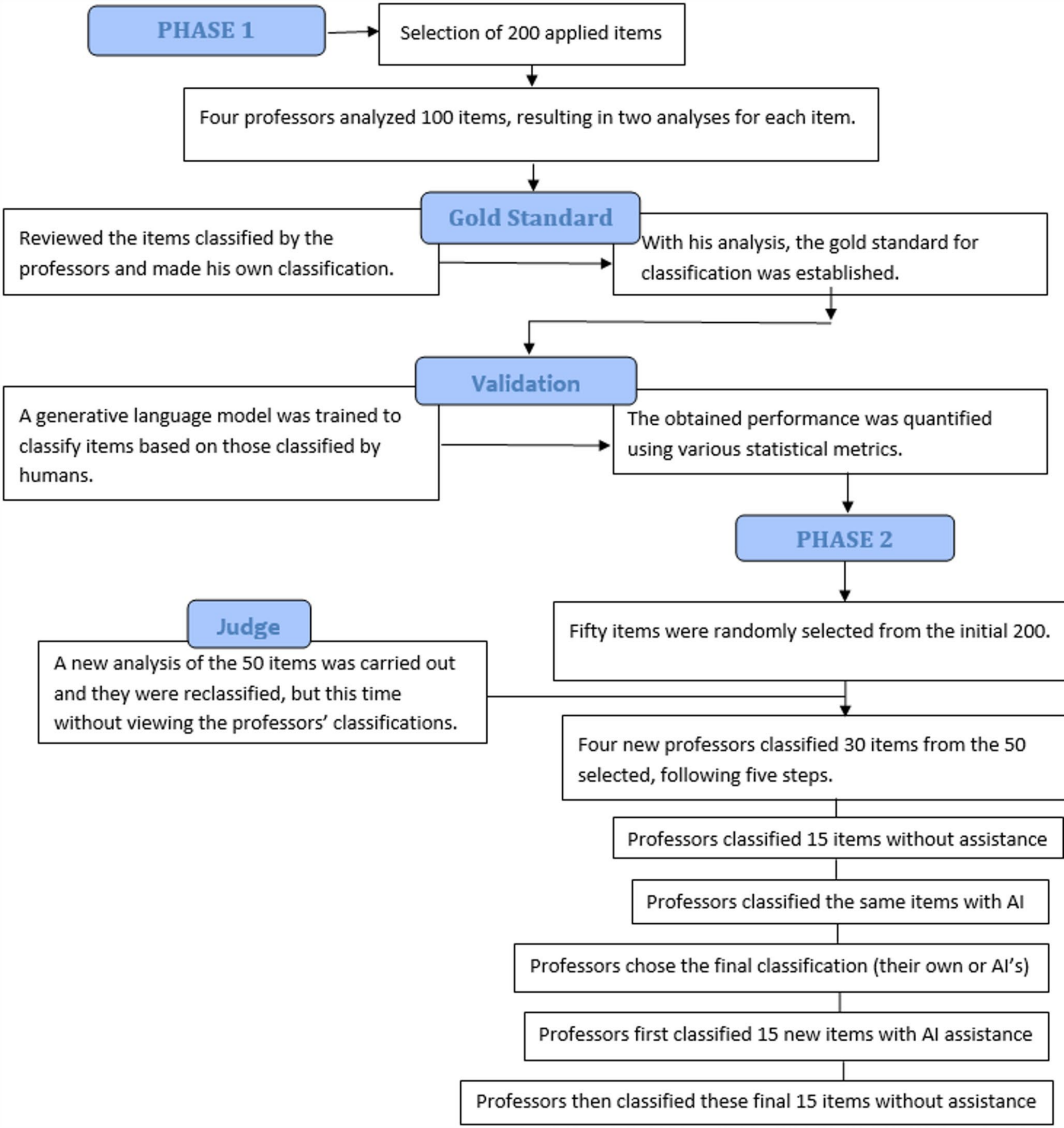
Methodological flowchart of item classification by bloom’s taxonomy

The following flowchart illustrates the study design (Fig. 1).

## Sample and Data Set

### PHASE 1

The test items were selected from the item database from the medical school of a University Center in Brazil, with the consent of the dean’s office. The medical course is structured in eight preclinical semesters, followed by four semesters of clinical internship.

Two hundred test items were selected, half from the first semester and half from the seventh semester. These items were selected at random, considering only the year of application (2021) and the semester (1st and 7th). The choice of items from the first and seventh semesters aimed to provide a varied sample of items in terms of cognitive skill complexity.

In this first phase, our PhD professors with extensive experience in health education assessment were considered experts in cognitive assessment were selected. They classified 100 items each according to the taxonomy, as they understood it. Thus, each item would be analyzed by two evaluators. We chose the option of only 100 for each evaluator so as not to be too exhaustive. The initial forecast was that this analysis would be completed in around 15 to 30 days.

After this first part of stage 1, a judge (specialist) on the subject was asked to analyze the results. With over 40 years of experience in education, this specialist, who leads this pedagogical support center, made a final classification based on the classifications already made by the initial evaluators. Thus, we ended up with three evaluations for each item, the most relevant being the last one, that was done by the judge (specialist), to define the gold standard. This process took around 16 days.

Two distinct data sets were used:

- Training Set: Spreadsheet containing items already classified as explained above according to modified Bloom’s taxonomy, including detailed explanations justifying each classification (e.g., identification, comprehension, application, analysis, evaluation, creation).
- Test Set: An independent database with 100 new items, previously classified by experts, for external validation of the model’s performance.

#### Classification Procedure

The classification of items was conducted exclusively by a generative language model (ChatGPT PRO 4) following these sequential steps:

- Initial Classification

An initial classification of the items was performed using direct interpretation of the statement and explanatory observations provided by the experts, achieving an initial accuracy of approximately 90%.

- Iterative Refinement

Multiple additional iterations were conducted to adjust and improve the model, based on a detailed analysis of the errors made in the previous step. Despite these attempts, the best accuracy achieved remained stable at 90%.

- Final Model Selection

The final model selected was the one that performed best (stable accuracy of 90%) during incremental refinement.

#### External Validation

The selected model was tested on an independent set of 100 items classified by experts to validate its generalization ability. The performance obtained on this external set was quantified by several statistical metrics described in the statistical analysis section.

## PHASE 2

The use of the GPT application generated with the model trained in this study was tested by medical school professors and experts in the revised Bloom’s taxonomy. Four health professors with extensive experience in the teaching-learning process and classroom practice were selected. Two were from the medical program, one worked in academic-operational support for *Lato sensu* programs and also served as a project coordinator at the State School of Public Health, and the other worked at the Pedagogical Support Center. In this phase, they analyzed the same items, but this time the distribution was random rather than sequential, using only 50 of the initial 200 items. Each professor analyzed 30 items in five stages:

- Stage 1 – Classification of 15 items according to their understanding and without consultation
- Stage 2 – Classification of these same item using the AI classifier
- Stage 3 – Choice of a final classification (preliminary or AI)
- Stage 4 – Classification of another 15 items using the classifier first
- Stage 5 – Analysis of these same item based on their understanding and without consultation

After completing the analyses, the evaluators responded to the SUS scale.

In parallel with this, the expert from the first phase was also invited to perform a new analysis of the 50 items that were previously selected to compose the 30 analyzed in this phase by each professor. The reduction in the number of items to 50 was intended to ensure greater quality and accuracy in the assessment, as well as to meet the judges’ own demands, who reported significant cognitive fatigue when analyzing 200 questions. Considering that the study design requires detailed and reflective human judgment (human-in-the-loop), maintaining a high number of items could compromise the consistency of responses and introduce fatigue-related bias. Thus, the reduction of the sample was a well-founded methodological decision, aimed at preserving the internal validity of the data and the reliability of the evaluation performed by the experts. The professors were intentionally not given specific training on interpreting AI. This was a deliberate choice to simulate a real-world “plug-and-play” scenario where faculty might use such a tool without a prior workshop, thereby testing the tool’s intuitive utility and the “automation bias” effect in a naturalistic setting.

The expert did not use the AI classifier and performed the classification based on his own interpretation.

At the end of each phase, the information that was made available to participants individually was compiled into a single spreadsheet to facilitate data analysis.

Table 1 presents an example of classification.

**Table 1:** Example of AI vs. Human Classification Logic

Item Content	Human Initial	AI Classification	Rationale
*Scenario*: “A 60-year-old male with history of CHF presents with dyspnea. Which of the following is the first-line diuretic therapy?”	Remember	Remember	**Agreement**: Item requires retrieval of a specific factual association (CHF -> Furosemide) without manipulating data.
*Scenario*: “A 45-year-old male has serum Na + 125, Urine Osm > 100, Urine Na + > 40. He is euvolemic. Comparing these values against the algorithm for hyponatremia, what is the most likely diagnosis?”	Understand	Analyze	**Disagreement (AI Correct)**: Faculty viewed this as ‘understanding’ the definition of SIADH. The AI correctly identified that the student must *differentiate* between causes and *examine* multiple data points (breaking down the lab profile) to reach a conclusion, fitting the ‘Analyze’ criteria.”

### Statistical Analysis

The statistical analysis of this study involved multiple metrics that allowed for accurate evaluation of the proposed model’s performance. Initially, the overall accuracy of the model was calculated on the independent set of 100 items previously classified by experts, resulting in 95%, indicating high overall performance. To detail this performance, a confusion matrix was constructed that clearly revealed the patterns of correct and incorrect answers in the specific categories of the modified Bloom’s Taxonomy (“Remember,” “Understand,” “Apply,” “Analyze,” “Evaluate,” and “Create”). From this matrix, the classic metrics of precision, sensitivity (recall), and F1-score were calculated for each category. Due to the presence of classes with different frequencies, sensitivity, specificity, and Matthews Correlation Coefficient (MCC) were also evaluated to adequately address the imbalance in the categories, confirming the previous results and reinforcing the diagnostic robustness of the model, especially in dominant classes. To verify the overall agreement between the classifications made by the model and the experts, Cohen’s Kappa index was calculated, considered very good, with a 95% confidence interval obtained via bootstrap. The stability of the estimates obtained was further confirmed by the bootstrap technique with 1,000 replications for overall accuracy. Finally, the McNemar test was used to test the statistical significance of the differences in the classifications made by the model compared to the expert classification, confirming high overall agreement of the results (despite statistical limitations due to the low error rate). Additionally, a detailed analysis of the errors was performed, revealing a predominance of confusion between the categories “Understand” and “Analyze,” highlighting specific areas for future improvement of the model. In summary, the statistical methods applied demonstrated the robustness of the estimates and significant accuracy of the classifications made by the model.

### Ethics

This research was conducted in accordance with the ethical principles for medical research involving human subjects outlined in the Declaration of Helsinki. All methods were carried out in accordance with relevant guidelines and regulations. The survey was approved by the Unichristus Research Ethics Committee (Comitê de Ética em Pesquisa da Unichristus) in Brazil (CAAE number 79520524.7.0000.5049). Written informed consent was obtained from all participants prior to their inclusion in the study.

## Results

The performance evaluation of the developed model revealed an overall accuracy of 95%, indicating that it correctly classified most items in the independent database of 100 items.

**Fig. 2:**
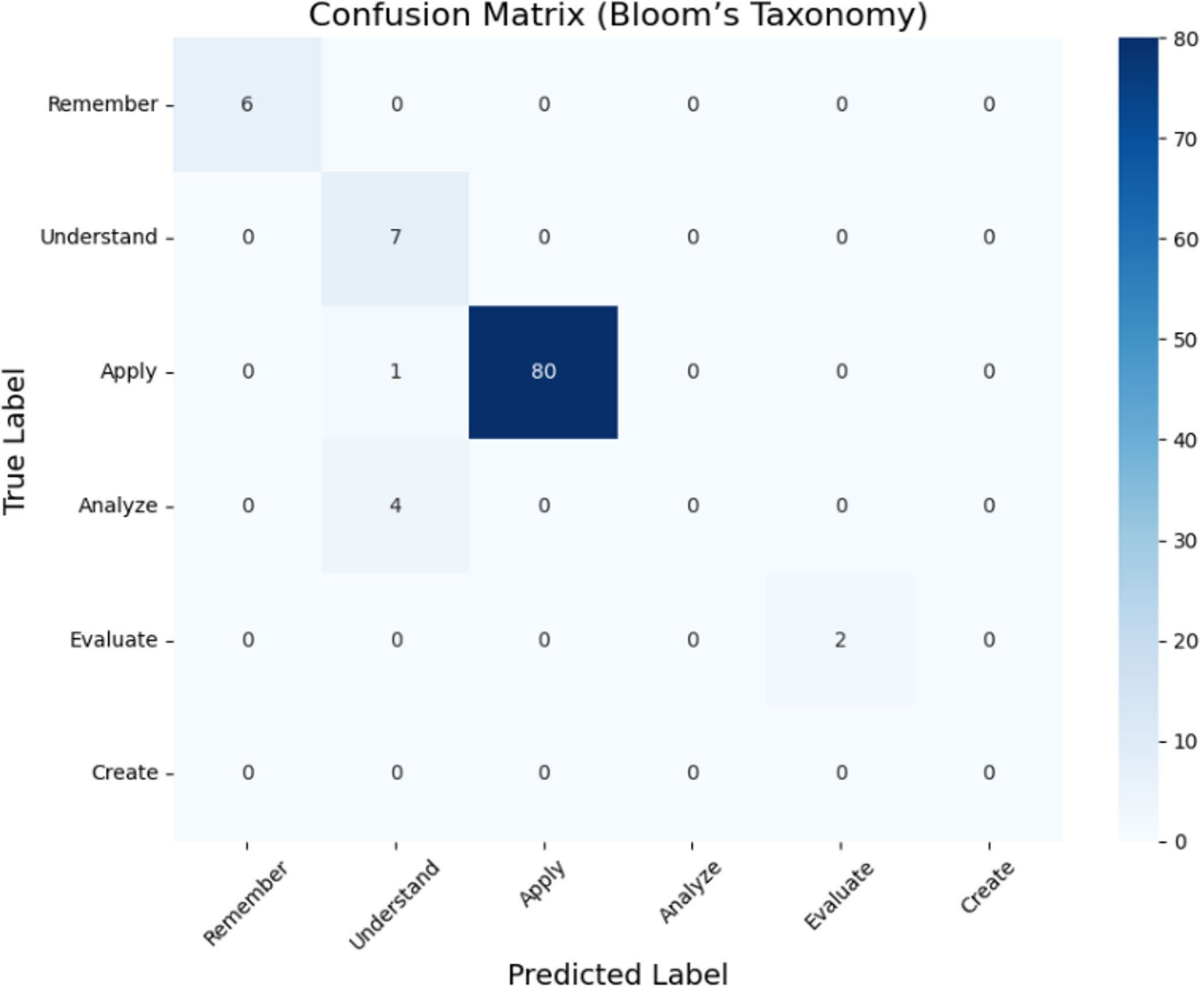
Confusion Matrix with Color Legend

As shown in the confusion matrix (Fig. 2), the model obtained particularly good results in the categories “Apply,” “Remember,” and “Evaluate,” with excellent precision, recall, and F1- scores close to or equal to 100%. The “Understand” category showed moderate accuracy, despite having high recall, indicating a certain tendency for the model to incorrectly classify items from other categories as belonging to this class. Notably, the “Analyze” category performed poorly, accounting for most of the errors made by the model.

Sensitivity, specificity, and the Matthews correlation coefficient (Table 2) confirmed these results, highlighting the model’s good performance in the categories with the highest prevalence in the analyzed set and revealing specific limitations in minority categories, especially in the “Analyze” category.

**Table 2:** Sensitivity, Specificity & MCC

Category	Sensitivity (Recall)	Specificity	MCC
Remember	10	10	10
Understand	10	9.46237E + 14	7.42948E + 15
Apply	9.87654E + 15	10	9.68644E + 15
Analyze	0	10	0
Evaluate	10	10	10
Create	0	10	0

The analysis of bias and prevalence of categories (Table 3) showed that the model did not simply reproduce the original distribution of data, indicating that it managed to capture discriminative characteristics of the item regardless of the frequency of categories.

**Table 3:** Bias & Prevalence

	Prevalence	Predicted Bias
Analyze	4	0
Apply	81	8
Evaluate	2	2
Understand	7	12
Remember	6	6

Detailed analysis of the errors (Table 4) confirmed that most confusion occurred between the categories “Understand” and “Analyze,” reflecting specific challenges in differentiating between these two categories.

**Table 4:** Error Taxonomy

True	Predicted	Count
Analyze	UNDERSTAND	4
Apply	UNDERSTAND	1
Remember	UNDERSTAND	0
Create	ANALYZE	0
Create	APPLY	0
Create	UNDERSTAND	0
Create	REMEMBER	0
Evaluate	CREATE	0
Evaluate	ANALYZE	0
Evaluate	APPLY	0
Evaluate	UNDERSTAND	0
Evaluate	REMEMBER	0
Analyze	CREATE	0
Analyze	EVALUATE	0
Analyze	APPLY	0
Analyze	REMEMBER	0
Remember	APPLY	0
Apply	CREATE	0
Apply	EVALUATE	0
Apply	ANALYZE	0
Apply	REMEMBER	0
Understand	CREATE	0
Understand	EVALUATE	0
Understand	ANALYZE	0
Understand	APPLY	0
Understand	REMEMBER	0
Remember	CREATE	0
Remember	RATE	0
Remember	ANALYZE	0
Create	EVALUATE	0

**Fig. 3:**
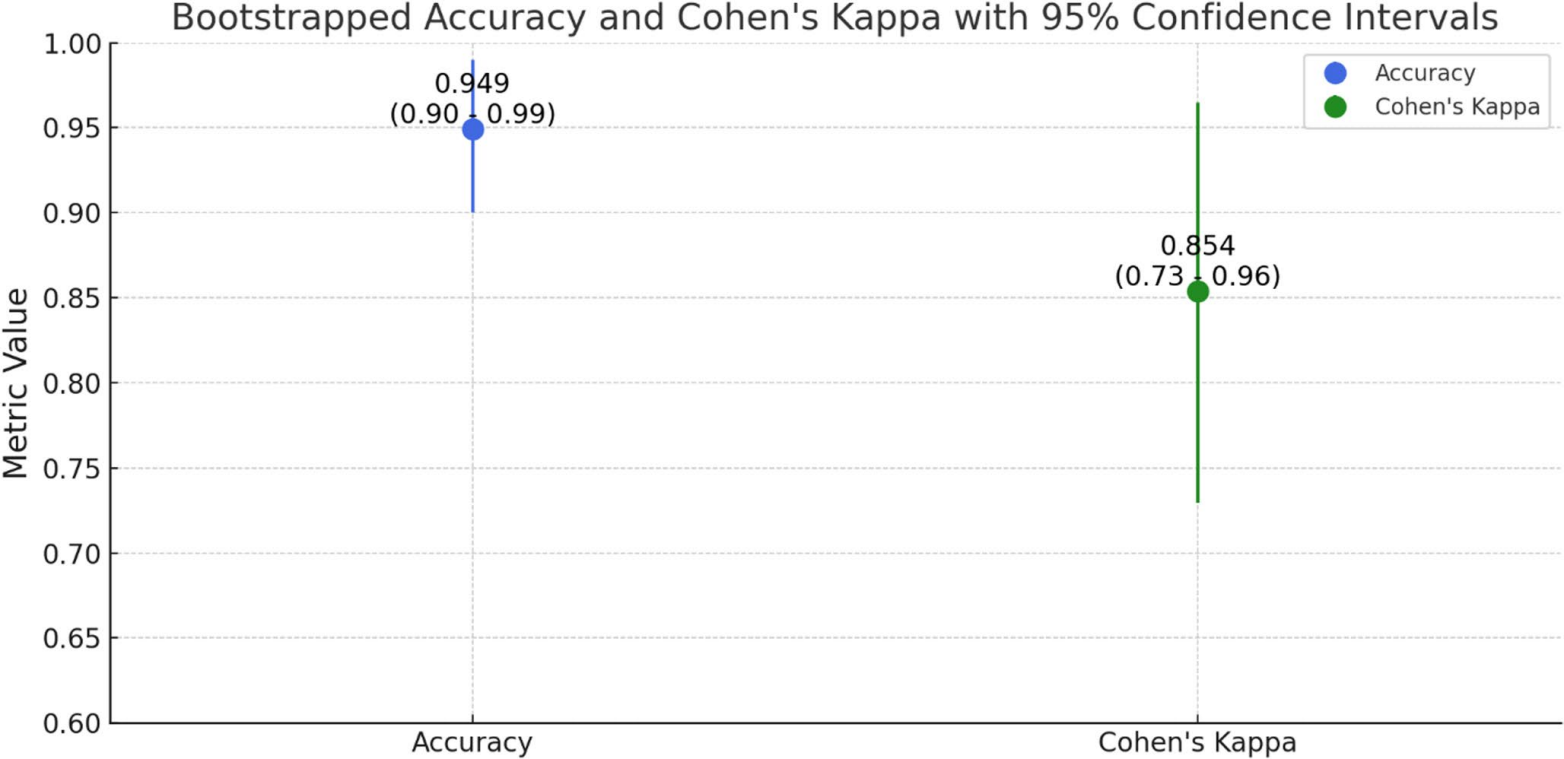
Bootstrapped Accuracy and Cohen’s Kappa with 95% Confidence Intervals

The robustness of the agreement between the classifications made by the model and those made by experts was confirmed using Cohen’s Kappa index (κ = 0.85), with a 95% confidence interval ranging from 0.73 to 0.96 (Fig. 3), indicating a high degree of agreement. In addition, statistical significance analysis using the McNemar test supported that the classifications obtained were consistent and robust. Finally, the stability of the model’s overall accuracy was confirmed using the bootstrap method, which provided a 95% confidence interval between 90% and 99%, corroborating the accuracy and reliability of the estimates obtained (Fig. 3).

The performance of two adjudicators was evaluated based on their agreement with an expert standard, their response to a conflicting artificial intelligence recommendation, and the ultimate accuracy of their decisions. Adjudicator 1 initially agreed with the expert standard in 45.0% of cases, whereas Adjudicator 2 agreed in 63.2% of cases. When presented with a conflicting AI recommendation in instances of initial disagreement, Adjudicator 2 changed their decision to align with the AI more frequently than Adjudicator 1 (57.1% vs. 22.7%, respectively). In cases where adjudicators maintained their original decision against both the expert standard and the AI recommendation, the AI’s assessment was ultimately determined to be correct in 70.6% of the cases for Adjudicator 1 and 66.7% for Adjudicator 2. A detailed breakdown of these findings is presented in Table 5.

**Table 5:** Comparison of adjudicator agreement with expert standard, adherence to AI recommendations, and accuracy in cases of disagreement

Characteristic	Adjudicator 1 *n* (%)	Adjudicator 2 *n* (%)
Initial assessment		
Total cases evaluated	40	19 (100.0)
Agreement with expert standard	18 (45.0)	12 (63.2)
Disagreement with expert standard	22	7 (36.8)
Response to AI in cases of initial disagreement		
Total cases with initial disagreement	22 (100.0)	7 (100.0)
Changed decision to align with AI	5 (22.7)	4
Maintained original decision	17	3
Accuracy analysis in cases of maintained disagreement		
Total cases with disagreement maintained	17 (100.0)	3 (100.0)
AI recommendation was correct	12 (70.6)	2 (66.7)
Adjudicator decision was correct	5 [17]	1

Percentages are calculated based on the total for each section (column percentages) and may not sum to 100 due to rounding.

The analysis of judge interactions with the artificial intelligence (AI) decision-support tool is summarized in Table 6. The median concordance between judges prior to the AI intervention was 54.1%. Following the presentation of the AI recommendation, a median change of 39.9% in initial judgments was observed. The total adherence rate to the AI’s final recommendation was 75.2%. For cases in which judges did not adhere to the AI recommendation, the median accuracy of their final judgment was 29.4%.

**Table 6:** Key metrics of judge interaction with the artificial intelligence decision-support tool

Metric	Value
Median pre-AI concordance	54.1
Median post-AI change	39.9
Total adherence to AI	75.2
Median accuracy of non-adherent judges	29.4

## Discussion

The evaluation of the model showed high overall performance, achieving 95% accuracy. The results showed high consistency in categories such as Remember, Apply, and Evaluate, with metrics close to 100% in precision, recall, and F1-score. In contrast, only moderate performance was observed in Understand and fragility in Analyze, responsible for most errors. Above all, the use by teachers demonstrated their adherence to AI suggestions and an increase in the accuracy rate in classification by AI-supported teachers compared to the expert.

This finding is especially relevant as it transcends the more common use of LLMs in medical education, which is predominantly focused on answering questions or supporting clinical learning, opening up a little-explored field: the automated curation of assessment items with psychometric rigor. The overall accuracy of 95% observed in this study is notably higher than that reported in many previous studies that evaluated automatic approaches to the classification of educational objectives or assessment test items. For example, despite multiple attempts to automate the classification of items according to Bloom’s Taxonomy, the highest accuracy obtained in previous studies did not exceed 93.5%, highlighting the need for further advances in this area [19]. Furthermore, even the best-performing deep learning models achieved only 86% accuracy. Similarly, previous studies adjusted a *Naïve Bayes* classifier to predict the taxonomy level and found that, in many cases, accuracy did not exceed 90%, reinforcing the methodological limitations reported thus far [20] .

In the field of automation applied to educational classification, a recurring pattern of reports on the fragility of model performance can be observed in the literature. Empirical evidence shows that systems based on machine learning and deep learning tend to have high accuracy at the lower levels of Bloom’s Taxonomy (Remember and Understand), but reveal a progressive decline at intermediate and higher cognitive levels, such as Apply, Analyze, Evaluate, and Create [13, 14]. More recent studies confirm this limitation, demonstrating that even in advanced architectures—such as *ensemble* models that integrate DistilBERT and TF-IDF—the difficulty in discriminating between adjacent categories persists. This finding suggests the existence of a structural limit of algorithms in the face of the intrinsically overlapping nature of taxonomy, reflecting the complexity of capturing gradual transitions between cognitive levels [19] .

In our results, we observe that, despite the high overall accuracy obtained, the analysis of the confusion matrix corroborated by the other statistical tests revealed a limitation common to both the model and the human evaluators: the difficulty in differentiating between the *“Understand”* and *“Analyze”* levels, which are already particularly challenging even for experienced specialists. This finding is consistent with the literature, which points to the tenuous nature of the boundaries between intermediate levels of taxonomy, often conditioned by the context and pedagogical intent of the item [17]. Furthermore, empirical studies show that inter-rater reliability in the classification of items according to Bloom’s Taxonomy tends to be low when all six categories are used, improving only when the levels are grouped into broader ranges of cognitive complexity [6]. This difficulty stems largely from the conceptual overlap between adjacent categories and the limitation of using only operational verbs as indicators of the cognitive process, since the actual demand of an item depends on the context and the type of knowledge mobilized [21] .

These findings reinforce our analysis that classification errors are concentrated at conceptual boundaries between adjacent levels, such as “Understand” and “Analyze.” On the other hand, the phenomenon described by “Bloom’s Taxonomy Inversion” [16] provides an interpretive framework for the unexpected results: AI systems, unlike humans, seem more suited to creative and evaluative tasks than to factual recall or the application of simple rules. This inversion, far from being an “error” to be corrected, reflects the statistical and generative nature of LLMs, which favors patterns of textual synthesis and recombination. Thus, while human teachers tend to ascend gradually through Bloom’s hierarchy, generative IAS can take the opposite path, imposing new pedagogical demands—especially in the sense of stimulating critical validation and refinement of algorithmic productions in students [22]. Corroborating these difficulties in classifying Bloom’s Taxonomy by AI, it was evident that the quality and pedagogical fidelity of items generated by LLMs strongly depend on the *prompting* strategy, with more elaborate combinations (e.g., inclusion of definitions and examples) improving adherence to Bloom’s levels but not eliminating inconsistencies.

This study innovated by promoting a triple comparison (AI × teachers × experts), allowing for an integrated assessment of both the model’s performance and its impact on teaching decisions. The comparison between teachers and experts allowed for an integrated assessment of both the model’s performance and its influence on teaching decisions. The evaluators’ analysis revealed significant differences in their behavior toward AI, suggesting that individual factors such as confidence in their own experience, receptivity to technology, and perception of AI authority can modulate human-machine interaction. In this context, it should not be forgotten that algorithmic recommendations can intensify cognitive biases, such as the *anchoring effect* and *automation bias*, leading to potentially misguided judgments. In this sense, Carter et al. systematically demonstrated that AI can anchor human decisions, inducing deference to the algorithm’s suggestions even when they do not represent the most appropriate choice. The authors also emphasize that factors such as the evaluator’s familiarity with the task and the transparency of the system can mitigate such biases, pointing to the need for a more critical and responsible use of artificial intelligence in evaluation processes [23]. 

The finding that faculty accuracy dropped to 29.4% when they disagreed with the AI is illuminating. It suggests a phenomenon of human overconfidence in the absence of objective feedback. This has profound practical implications for the implementation of AI in medical education. We propose that such AI tools should not be viewed as ‘auto-graders’ but as ‘adversarial partners’ in the item writing process.

Institutions should consider implementing a ‘Human-in-the-Loop’ verification protocol. Under this model, high agreement between Human and AI (e.g., on ‘Remember’ items) allows for rapid processing. However, instances of disagreement, particularly in the ‘Understand vs. Analyze’ boundary, should trigger a mandatory metacognitive pause. Faculty should be required to document the pedagogical rationale for their classification when overriding the AI. This process disrupts automation bias and turns the assessment creation process into a continuous faculty development opportunity, ensuring that the ‘Scoring Inference’ of the exam remains valid.

Despite this initial divergence, it is observed that in both evaluators, when they decided to maintain their position against AI and against the expert, AI proved to be correct in most cases. These results reinforce the difficulty humans have in classifying assessment test items according to Bloom’s Taxonomy, partly due to the absence of formal pedagogical training and the subjective nature of the task. In relation to medical courses, it is observed that many medical teachers do not have formal pedagogical training, which, as a result, tends to limit the construction of items to more elementary cognitive levels [4, 5]. Thus, as highlighted in the specialized literature, ensuring the quality of assessments requires rigorous review processes and alignment between educational objectives, cognitive processes, and assessment strategies [7] .

On the other hand, AI intervention caused a significant change in decisions, leading three out of four final judgments to align with the model’s recommendation. This application can contribute to the improvement of the teaching-learning process, since tests based on this taxonomy have greater content validity and allow for a fairer and more consistent assessment of medical skills, covering clinical reasoning, decision-making, and other skills fundamental to professional training [24] .

This study has limitations that should be considered. The analysis was conducted based on a restricted database of items, which may limit the generalization of results to different areas of medical knowledge or broader educational contexts. In addition, validation was performed in an experimental environment, not fully reflecting the complex dynamics of classroom use or highly relevant assessments. Another point to highlight is that biases associated with both model training and human interpretations may have influenced the findings, requiring caution in extrapolating conclusions.

In summary, automated grading appears to be a promising strategy, capable of reducing teachers’ workload, increasing reliability, and, at the same time, improving the pedagogical quality of health assessments. Thus, this article makes an original contribution by demonstrating that automated assessment of the complexity of cognitive test items using generative language models is not only technically feasible but also educationally relevant, with the potential to improve the quality and equity of assessments in medical education.

By positioning generative AI as a critical partner in the educational process, that is, as a support tool and not as a substitute for the teacher’s critical thinking, the research paves the way for new investigations into its integration into medical curricula and teacher training programs, broadening the debate on how to balance technological innovation, pedagogical validity, and professional autonomy.

## Data Availability

The datasets used and/or analyzed during the current study are available from the corresponding author upon reasonable request.

## Abbreviations

PBL: Problem-Based Learning
hPBL: Hybrid Problem-Based Learning
